# Whole-genome analysis and phenotypic antimicrobial resistance of *Staphylococcus aureus* isolated from foods in Northern Kazakhstan

**DOI:** 10.3389/fmicb.2026.1910820

**Published:** 2026-08-07

**Authors:** Saniya S. Kozhakhmetova, Elena V. Zholdybayeva, Diana A. Basharova, Ayazhan Bekbayeva, Aiganym B. Toleuzhanova, Asset Zh. Daniyarov, Aitbay K. Bulashev

**Affiliations:** 1 National Scientific Shared Laboratory of Biotechnology, National Center for Biotechnology, Astana, Kazakhstan; 2 National Laboratory Astana, Center for Life Sciences, Nazarbayev University, Astana, Kazakhstan; 3 Department of General Biology and Genomics, L. N. Gumilyov Eurasian National University, Astana, Kazakhstan; 4 Department of Animal Husbandry and Biological Sciences, Institute of Animal Science and Veterinary Medicine, Saken Seifullin Kazakh Agrotechnical University, Astana, Kazakhstan

**Keywords:** antibiotic resistance genes, MLST, MRSA, staphylococcal toxins, *Staphylococcus aureus*, virulence factors

## Abstract

Staphylococcal food poisoning is widespread, causing significant public health problems. This study aimed to characterize the genomic profiles and antimicrobial susceptibility of *Staphylococcus aureus* isolates obtained from food products in Northern Kazakhstan using whole-genome sequencing. The study included 42 food-derived isolates from raw food products (milk, chicken fillet, beef, horse meat, semi-finished meat products) and ready-to-eat (RTE) products (sour cream, butter, canned tuna, culinary dishes). All isolates were recovered from individual samples collected between June 2024 and June 2025 in Northern Kazakhstan (Astana and Kostanay regions). They were characterized using antibiotic susceptibility testing, whole-genome sequencing, multilocus sequence typing (MLST), and analysis of resistance, enterotoxin, and virulence genes. Phenotypic resistance to cefoxitin (MRSA phenotype) was detected in 21.4% (9/42) of food-derived isolates; all corresponding strains carried the *mecA* gene associated with methicillin resistance. Multidrug resistance (MDR) was identified in 9.5% (4/42) of food-derived isolates. The predominant ST type among MRSA strains was ST22 (8 of 9 strains). Only a single strain, Sa847_KZ, has a truly indeterminate/unresolved profile. Among classical enterotoxin genes, *sea* was detected in 4.8% (2/42) and *sec* was detected in 9.5% (4/42) of food-derived isolates. Non-classical enterotoxin genes were more prevalent: *seg* was found in 57.1% (24/42) and *sei* in 19.0% (8/42) of food-derived samples. The *tsst* gene, encoding a toxin capable of causing severe acute illness, was detected in 28.6% (12/42) of food-derived isolates - a prevalence substantially higher than reported for the *tsst* gene in food-derived *S. aureus* from most other regions worldwide. The results emphasize the need for enhanced food quality control and monitoring of antimicrobial resistance in Northern Kazakhstan to prevent cases of food poisoning.

## 1 Introduction

*Staphylococcus aureus* is a gram-positive, facultatively anaerobic coccus. Unlike many other foodborne bacteria, it can survive under diverse environmental conditions, including high salt concentrations, a wide temperature range, and desiccation. Combined with its extensive repertoire of virulence factors, these characteristics make *S. aureus* one of the leading causes of foodborne illness worldwide. According to the World Health Organization, unsafe food causes approximately 600 million cases of foodborne illness annually, resulting in the loss of 33 million healthy life-years (Rodríguez-Melcón et al., 2024). Staphylococcal food poisoning (SFP), caused by the heat-stable enterotoxins of *S. aureus*, is one of the most common causes of bacterial foodborne intoxication worldwide and differs from other foodborne diseases in one important aspect: the thermal stability of these toxins allows them to retain biological activity even after bacterial cells have been inactivated during the heat treatment of food products (Shalaby et al., 2024; Govari and Pexara, 2025).

Considering the expected increase in deaths from antibiotic-resistant infections to 10 million cases by 2050 – 47.3% of which will occur in the Asian region – the epidemiological monitoring of *S. aureus* in food products is of strategic importance for global public health (Liang et al., 2023).

The exceptional physiological plasticity of *S. aureus* – including tolerance to high salt concentrations, a wide growth temperature range, and resistance to desiccation and osmotic stress – enables this pathogen to colonize virtually every segment of the food chain: from primary raw material processing and livestock slaughter to retail distribution and culinary preparation (Zhao et al., 2025; Alkuraythi et al., 2024). A systematic review and meta-analysis (Léguillier et al., 2024) established the overall prevalence of *S. aureus* in various food categories as follows: 35.1% in ready-to-eat products, 21.7% in meat products, and 18.5% in dairy products, with beef accounting for 44% of all contaminated meat products. Among dairy matrices, raw milk consistently acts as the primary reservoir: Zhang et al. (2022) reported an overall prevalence of 33.36% in raw milk based on data from 140 global studies spanning three decades; Shalaby et al. (2024) documented an intra-herd prevalence of enterotoxigenic *S. aureus* in raw ruminant milk of 11.6% in a systematic review. As established for Kazakhstan, milk is the most common source of foodborne *S. aureus* isolates (Baymenov et al., 2023).

The accelerating development of antimicrobial resistance (AMR) in foodborne strains of *S. aureus* is a particular cause for concern from a public health perspective. Resistance to penicillins, mediated primarily by the blaZ gene encoding staphylococcal β-lactamase, represents the most common resistance phenotype globally, recorded in 61% of all food isolates, with 68% of all isolates exhibiting resistance to at least one clinically relevant antibiotic (Léguillier et al., 2024). More disturbing is that methicillin-resistant *S. aureus* (MRSA) – identified by the presence of the mecA gene, which encodes the penicillin-binding protein PBP2a, has moved beyond its historical association with healthcare settings and become firmly established in the food chain. A systematic review and meta-analysis by Xing et al. (2025) established the overall prevalence of MRSA in meat and meat products at 3.72%. A review of 186 publications on *S. aureus* in milk and dairy products confirmed the detection of MRSA in 68.8% of studies (González-Machado et al., 2024).

Genomic analysis of 1,152 complete *S. aureus* genomes revealed an increase in the proportion of AMR-positive genomes from 88.68% in 2020 to a peak of 98.43% in 2024, reflecting an alarming escalation of the global resistome (Durairaj et al., 2025). A key factor in the spread of genetic determinants of resistance – including *mecA* (methicillin), *blaZ* (penicillins), *erm* (macrolides), *tet* (tetracyclines) and efflux pump genes – is played by mobile genetic elements: plasmids, transposons, staphylococcal chromosomal cassettes and pathogenic islands, which facilitate the horizontal transfer of resistance determinants both within species and between different bacterial species (Mlynarczyk-Bonikowska et al., 2022; Alkuraythi et al., 2024).

The pathogenic potential of *S. aureus* is determined by a broad spectrum of virulence factors, including classical staphylococcal enterotoxins types A-E (*sea-see*), non-classical enterotoxins (*seg, sei*), and numerous enterotoxin-like proteins (SEls), as well as hemolysins (α-, β-, δ-, γ-toxins), leukocidins (LukED, LukMF’, Panton-Valentine leukocidin (PVL)), and the superantigen toxic shock syndrome toxin-1 (TSST-1) (Tam and Torres, 2019). Staphylococcal enterotoxins pose a significant risk to food safety, as they are resistant to heat treatment, low pH conditions, and proteolytic degradation by digestive enzymes. Therefore, food products may remain toxic even after heat treatment that destroys viable bacterial cells (Shalaby et al., 2024). Although classical SEA has historically been considered the most frequently implicated toxin in staphylococcal food poisoning (SFP) outbreaks, global genomic-epidemiological data indicate a growing prevalence of non-classical enterotoxins, particularly SEG and SEI, among food isolates (Chen et al., 2023). Toxic shock syndrome toxin-1 (TSST-1), a potent superantigen capable of causing toxic shock syndrome, is increasingly detected in food-associated *S. aureus* strains, predominantly belonging to the ST22 epidemic clonal complex, indicating a convergence of antibiotic resistance and hypervirulence in food chains (Touaitia et al., 2025).

Bacteriophages (phages) exert the most significant impact on the diversity and evolution of *Staphylococcus aureus*. As mobile genetic elements, they actively participate in horizontal gene transfer, allowing strains to rapidly acquire novel virulence factors and antimicrobial resistance genes. In foodborne isolates, the presence of these phage-encoded elements not only enhances bacterial survival under processing stresses but also poses a serious risk for the dissemination of pathogenicity traits along the food chain (Ingmer et al., 2019). In contrast, the CRISPR/Cas system provides adaptive immunity against invading bacteriophages and plasmids, thereby limiting the acquisition of foreign genetic material (Jore et al., 2012). The balance between phage-driven acquisition and CRISPR-mediated restriction therefore influences how readily food-borne lineages accumulate resistance and virulence genes – which is why we screened our isolates for both prophage regions and CRISPR-Cas loci.

Whole-genome sequencing (WGS) has become the gold standard for comprehensive monitoring of foodborne *S. aureus*, enabling simultaneous, high-resolution profiling of AMR genes, virulence determinants, enterotoxin repertoires, and phylogenetic relationships within a single analytical workflow (Vingadassalon et al., 2025). Unlike traditional PCR methods or phenotypic susceptibility testing, WGS enables complete resolution of clonal lineages through multilocus sequence typing (MLST), phylogeny based on single-nucleotide polymorphisms (SNPs), and population structure analysis, which makes it possible to attribute outbreak strains to specific food products and identify cross-sector transmission. The WHO has officially recommended WGS as a priority tool for strengthening national foodborne disease surveillance systems as part of the Global Food Safety Strategy for 2022–2030 (WHO, 2022). Despite its growing adoption in high-income countries, whole-genome sequencing (WGS) of foodborne *S. aureus* remains significantly underrepresented in Central Asia and the post-Soviet region, creating a substantial geographical gap in the global AMR surveillance system.

In Kazakhstan, published data on *S. aureus* in food products are largely limited to phenotypic studies and PCR-based analyses of individual resistance genes Baymenov et al. (2023) characterized phenotypic antibiotic resistance in strains isolated from cattle milk in Northern Kazakhstan, while Rychshanova et al. (2022) investigated resistance genes in dairy-associated strains using PCR. While these studies provide valuable baseline data, they are inherently limited by the absence of genomic information, which restricts their utility for tracking clonal lineages, comprehensively characterizing the resistome and virulome, and assessing the potential for horizontal gene transfer. To our knowledge, no WGS studies of foodborne *S. aureus* have been conducted in Northern Kazakhstan to date, despite the country’s significant livestock sector and its role as a major producer of dairy products and meat. This gap is particularly significant given Central Asia’s geographical location at the crossroads of European, Russian, Chinese, and South Asian AMR epidemiology, which suggests the region’s potential role in the cross-border spread of resistant clones.

This study aimed to comprehensively characterize the genomic profiles and phenotypic antimicrobial susceptibility of *S. aureus* isolates obtained from food products in Northern Kazakhstan.

## 2 Materials and methods

### 2.1 Research objects

A total of 181 individual food samples were collected between June 2024 and June 2025 in Northern Kazakhstan (Astana and the Kostanay regions) and screened for *Staphylococcus aureus*. The samples comprised 38 raw milk samples, 65 dairy products, 19 chicken meat samples, 18 beef samples, 3 horse meat samples, 3 fish samples, 3 pork samples, 4 egg samples, 13 semi-finished food products, and 15 ready-to-eat culinary dishes. Samples were obtained by convenience sampling from farms and retail stores. The 42 food-derived isolates were recovered from two categories of products: (i) Raw food products (*n* = 33), including raw cow’s milk (*n* = 16) and raw mare’s milk (*n* = 1); raw chicken fillet (*n* = 9); raw beef (*n* = 2); raw horse meat (*n* = 1); and semi-finished raw meat products (minced beef and frozen beef dumplings, *n* = 4); (ii) Ready-to-eat (RTE) food products (*n* = 9), including dairy products intended for direct consumption (sour cream, *n* = 4; butter, *n* = 1); canned tuna (*n* = 1); and ready-to-eat culinary dishes (Olivier salad, Alfredo pasta, and cream puffs; *n* = 3). To ensure consistency and avoid overrepresentation, each isolate was recovered from a separate positive sample (1:1 ratio).

**FIGURE 1:**
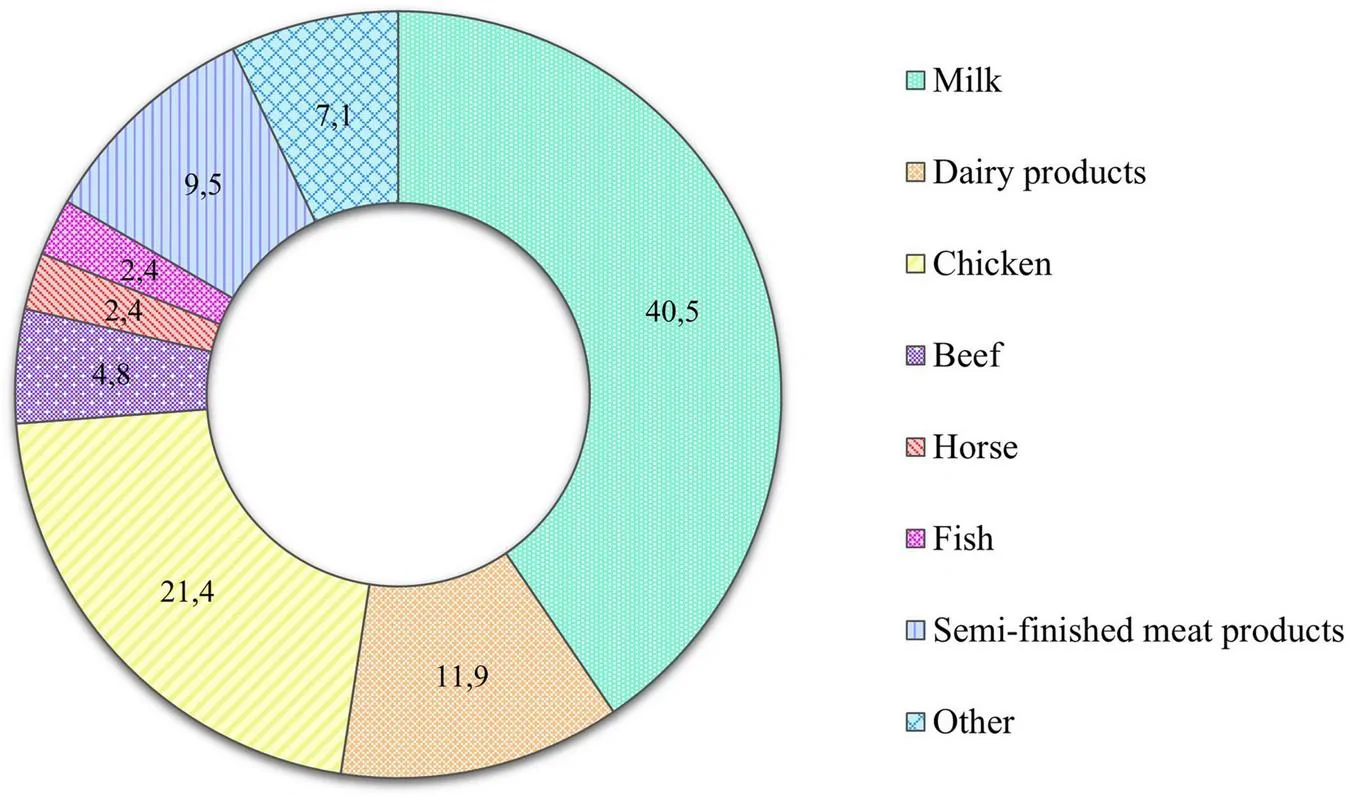
Prevalence of *S. aureus* strains in food products, % (*n* = 42).

For the analytical grouping used in Figures 1, 4, 6–9 food-derived isolates were assigned to one of eight categories according to the matrix of origin: (1) milk – raw cow’s and mare’s milk (*n* = 17); (2) dairy products - sour cream and butter (n = 5); (3) chicken meat – raw chicken fillet (*n* = 9); (4) beef - raw beef (*n* = 2); (5) horse meat – raw horse meat (*n* = 1); (6) fish – canned tuna (n = 1); (7) semi-finished meat products – minced beef and frozen beef dumplings (*n* = 4); and (8) “Other” (*n* = 3), defined as ready-to-eat composite culinary dishes (Olivier salad, Alfredo pasta, and cream puffs) in which *S. aureus* could originate from any one of several ingredients or from cross-contamination during preparation.

The samples were transported in sealed containers at a temperature of +++4 °C using refrigerants. Microbiological analysis was performed within 24 h after collection.

### 2.2 Isolation and identification of *S. aureus*

*S. aureus* was isolated from food samples using standard microbiological methods. Liquid culture media were used for primary enrichment: nutrient broth, Luria-Bertani (LB) broth (HiMedia, Mumbai, India). Meat peptone agar and Luria-Bertani agar (HiMedia, Mumbai, India) were used as solid media for culture.

Selective isolation of *S. aureus* was performed using chromogenic media: Chromatic *Staph. aureus* chromogenic agar (Liofilchem, Teramo, Italy) (chromogenic substrates in the medium are broken down by *S. aureus* enzymes, forming purple colonies, which allows it to be visually distinguished from other staphylococci). In addition, Baird–Parker agar was used for selective isolation. Due to the presence of selective agents (lithium and tellurite) and differential components (egg yolk, glycine, and pyruvate), typical *S. aureus* colonies appear black with clear halos. It should be noted that this medium is used to detect coagulase-positive staphylococci, in particular *S. aureus*, allowing a double effect to be observed: black colonies (reduction of potassium tellurite to metallic tellurium) and a transparent zone around the colonies (lipolytic and proteolytic activity of staphylococci, destroying egg yolk). From the total samples, 42 food-derived isolates were recovered based on these selective characteristics.

Final identification at the species level was performed using the MALDI Biotyper system equipped with a microflex LT mass spectrometer (Bruker Daltonik GmbH, Bremen, Germany). Freshly grown bacterial colonies were applied to the spots of a 96-well polished steel target plate (MSP 96 target polished steel BC, microScout Target). Each spot was then overlaid with 1 μL of matrix solution (saturated -cyano-4-hydroxycinnamic acid (HCCA) in 50% acetonitrile and 2.5% trifluoroacetic acid (TFA)) and air-dried at room temperature. Mass spectra were acquired in linear positive mode within a mass range of 2,000–20,000 Da. The instrument was calibrated using the Bacterial Test Standard (BTS) (Bruker Daltonics). Identification was performed using MALDI Biotyper RTC Version 4.0 (Build 11) software (Bruker Daltonics) and compared against the BDAL-12.0.0.0 reference library (11897 MSP). A log(score) of 2.0 was used as the threshold for reliable species-level identification.

Hemolytic activity was evaluated by streaking each isolate onto Columbia Agar Base (Liofilchem, Teramo, Italy) supplemented with 5% defibrinated sheep blood (Ecolab, Russia). Plates were incubated aerobically at 37 °C for 24 h. Hemolytic activity was assessed by visual examination of the zone surrounding bacterial colonies and classified as β-hemolysis (complete clearing of erythrocytes), α-hemolysis (partial hemolysis producing a greenish discoloration), or γ-hemolysis (absence of hemolysis), according to standard clinical microbiology procedures (Abebe et al., 2024; Bannerman, 2003).

DNA extraction was performed using Cetyl Trimethyl Ammonium Bromide (CTAB)/NaCl (Wilson, 2001). The quantitative characteristics of total DNA were measured spectrophotometrically using a Nanodrop 1000 Spectrophotometer (Thermo Fisher Scientific, Wilmington, USA) at a wavelength of 260 nm. For a more accurate determination of DNA concentration, a Qubit 2.0 fluorometer (Invitrogen/Life Technologies, Carlsbad, USA) was used. The qualitative characterization of total DNA was performed by electrophoresis in 1% agarose gel (Applichem, Darmstadt, Germany).

The 16S rRNA gene fragment was amplified using universal bacterial primers: 8F (5′-AGAGTTTGATCCTGGCTCAG-3′) (Edwards et al., 1989) and 806R (5′-GGACTACCAGGGTATCTAAT-3′) (Caporaso et al., 2011). The PCR reaction (total volume 20 μl) contained: 1x Taq buffer; 2.5 mM MgCl; 0.2 mM of each dNTP; 1 U of Taq DNA polymerase; 10 ng of each primer; 100 ng of template DNA.

The PCR reaction (total volume 20 μl) contained: 1x Taq buffer; 2.5 mM MgCl; 0.2 mM of each dNTP; 1 U of Taq DNA polymerase; 10 ng of each primer; 100 ng of template DNA.

The amplification was performed using the following thermal profile: initial denaturation at 95 °C for 10 min; 35 cycles of denaturation at 95 °C for 30 s, annealing at 55 °C for 30 s, and extension at 72 °C for 60 s; followed by a final extension at 72 °C for 7 min. The resulting PCR products were purified and sequenced using an ABI 3730xl Genetic Analyzer (Applied Biosystems, USA). The resulting sequences were analyzed using the BLAST algorithm against the GenBank database.

### 2.3 Determination of sensitivity to antimicrobial drugs

The sensitivity of *S. aureus* isolates to antimicrobial drugs was determined using the OCHA Sensilla Test Staphy kit (Erba Lachema, Czech Republic).

Testing was conducted using the broth microdilution method in accordance with the manufacturer’s instructions. The study included 12 antimicrobial drugs belonging to various pharmacological classes: β-lactams (penicillin, cefoxitin), macrolides (erythromycin), lincosamides (clindamycin), oxazolidinones (linezolid), phenicols (chloramphenicol), tetracyclines (tetracycline), fluoroquinolones (ciprofloxacin), folate pathway antagonists (trimethoprim/sulfamethoxazole), aminoglycosides (gentamicin), glycopeptides (vancomycin), and nitrofurans (nitrofurantoin).

The minimum inhibitory concentrations (MICs) of antibiotics were determined after incubation at 35 °C for 20 h under aerobic cultivation conditions. This temperature was chosen because cultivation at 35 °C is necessary to detect staphylococci resistant to cefoxitin, and to take into account the results of vancomycin susceptibility testing, incubation was carried out for 24 h. Interpretation was performed in accordance with the current clinical breakpoints of the European Committee on Antimicrobial Susceptibility Testing (EUCAST v.16.0 dated January 1, 2026) for *S. aureus^1^* . Quality control of the testing was performed using the reference strain *S. aureus* ATCC 29213.

Isolates of *S. aureus* resistant to three or more classes of antimicrobial agents were classified as multidrug-resistant (MDR) according to international criteria proposed by Magiorakos et al. (2012).

### 2.4 Calculation of the multiple antibiotic resistance (MAR) index

The MAR index was calculated and interpreted in accordance with the methodology of (Krumperman, 1983) according to the formula:


MAR=a/b,

where “a” refers to the number of antibiotics to which the isolate was resistant, and “b” refers to the total number of antibiotics tested.

### 2.5 Whole-genome sequencing and bioinformatic analysis

DNA samples were prepared for sequencing using Collibri™ PS DNA Library Prep Kits for Illumina Systems (Invitrogen/Life Technologies, USA) and MiSeq^®^ 600 cycles PE Reagent Kit v3 (Illumina, USA) according to the manufacturer’s instructions. Sequencing was performed on an Illumina MiSeq instrument (Illumina, San Diego, USA).

Quality control of raw sequencing reads was performed using FastQC v0.11.9 (Babraham Bioinformatics, Cambridge, UK). Low-quality bases and adapter sequences were trimmed using Trim Galore v0.6.5 (Krueger, Babraham Bioinformatics), which internally invokes Cutadapt for adapter removal. Read quality was verified by a second round of FastQC after trimming.

De novo genome assemblies were generated from paired-end short reads using Unicycler v0.4.8 (Wick et al., 2017), with a minimum contig length threshold of 300 bp and minimum contig coverage of 5×. Assemblies were polished through two iterative rounds of Pilon (Walker et al., 2014) using Bowtie2^2^ v2.4.4 (Langmead and Salzberg, 2012) for read mapping and SAMtools v1.20 for BAM processing. Assembly quality metrics - including total assembly length, number of contigs, N50, and GC content - were evaluated using QUAST v5.2.0 (Gurevich et al., 2013).

Taxonomic identity and primary genome annotation of assembled genomes were performed using the RAST toolkit (RASTtk; Brettin et al., 2015). Annotation included identification of protein-coding sequences (CDS), transfer RNA (tRNA), and ribosomal RNA (rRNA) genes, as well as functional assignments based on FIGfam protein families. Plasmid content was assessed as part of the annotation pipeline; no plasmid replicons were detected in the studied isolates.

Statistical analysis was performed using Microsoft Excel, and Pearson correlation coefficients (\(*r*\)) were determined to assess the correlation between variables.

### 2.6 Molecular typing and phylogenetic analysis

MLST was performed using MLST v2.23.2 (Seemann, 2014) against the PubMLST^3^ *S. aureus* scheme based on seven housekeeping loci (*arcC, aroE, glpF, gmk, pta, tpi, yqiL*). Sequence types were assigned based on allelic profiles.

Raw paired-end sequencing data from six isolates (Sa829, Sa142, Sa845, Sa846, Sa847, Sa2206) were further analyzed using SRST2 (v[0.2.0]) to identify MLST profiles that could not be identified after assembly-based typing. The analysis was run using the MLST allele sequences of *Staphylococcus aureus*. SRST2 maps sequencing sequences directly to reference alleles at a locus and reports the best-matching allele, the number of mismatches relative to this allele, and the average read depth across the entire locus for each of the seven MLST loci. SRST2 was run with default parameters. For each isolate, the resulting seven-locus allelic profile was used to assign a sequence type (ST) according to the *S. aureus* MLST scheme, and the results were compared with the corresponding assembly results. Isolates with exact (zero mismatches) allelic matches at all seven loci were assigned to specific sequence types (STs). Isolates with a single-nucleotide mismatch at one locus relative to the closest reference allele were assigned to putative sequence types (STs) or ST-like profiles.

Core genome SNP-based phylogeny was inferred using CSI Phylogeny v1.4 (Kaas et al., 2014). SNPs were called with the following filters: minimum depth 10×, minimum relative depth 10%, SNP pruning distance 10 bp, minimum SNP quality (Phred) 30, minimum mapping quality 25, and Z-score threshold 1.96. Maximum likelihood phylogenetic trees were constructed from concatenated SNP alignments and visualized with metadata annotations.

goeBURST analysis was performed to identify clonal complexes and assess population structure. Allelic distances between sequence types were calculated across the seven MLST loci, and network graphs were constructed connecting STs differing by ≤5 alleles. Visualization was performed in R v4.4.2 using tidyverse (Wickham et al., 2019), igraph (Csardi and Nepusz, 2006), and ggraph (Pedersen, 2024) packages with the Fruchterman-Reingold layout algorithm.

### 2.7 Antimicrobial resistance and virulence gene analysis

AMR genes were identified from genome assemblies and screened for determinants conferring resistance to β-lactams (blaZ, mecA), macrolides (ermA, ermC), fluoroquinolones (norA, norC), tetracyclines (tetK, tetM), and chloramphenicol (cat(pC221)). Gene presence/absence was determined using the Comprehensive Antibiotic Resistance Database (CARD)^4^ and ResFinder^5^. CARD analysis was performed using the Resistance Gene Identifier (RGI) v6.0.5 web portal with CARD v4.0.1. Open reading frames were predicted using Prodigal, homologous sequences were identified with DIAMOND, and resistance genes were assigned using the Perfect criterion (100% sequence identity over the matching region) under the Strict detection model based on the curated CARD bit-score cut-offs. For ResFinder, antimicrobial resistance genes were identified using version 4.7.2 with a minimum sequence identity threshold of 99%. Virulence factors were identified using the Virulence Factor Database (VFDB)^6^. Draft genome sequences in raw FASTA format were uploaded for analysis. Coding sequences (CDSs) were automatically predicted by the integrated GLIMMER3 algorithm before virulence gene identification. No user-defined threshold parameters (e.g., sequence identity or coverage) were applied, as the VFanalyzer web interface does not provide this option; analyses were conducted using the default settings. For the present study, only the Toxins category of the VFanalyzer output was included in the analysis and reported in the manuscript. Additionally, the CRISPRCasFinder tool was utilized to identify the presence of CRISPR/Cas systems and arrays of spacers within the investigated genomes (Couvin et al., 2018). The prophage regions within the genome assemblies were predicted and annotated using the PHASTEST web server (Grant et al., 2023).

## 3 Results

### 3.1 Prevalence of *S. aureus* in foods

Of the 181 food samples screened during the study, *S. aureus* was recovered from 42, corresponding to an overall prevalence of 23.2% (42/181) in the food matrices examined from Northern Kazakhstan. In line with the 1:1 sample-to-isolate design (see Section “2.1 Research objects”), each of the 42 positive food samples yielded a single unique *S. aureus* isolate, which was taken forward for phenotypic and genomic characterization.

Figure 1 shows the distribution of the 42 positive food samples across product categories. Milk was the most frequently contaminated food matrix, accounting for 17/42 positive samples (cow’s milk, *n* = 16; mare’s milk, *n* = 1), followed by raw chicken meat (9/42), semi-finished raw meat products (4/42), dairy products intended for direct consumption (sour cream, *n* = 4; butter, *n* = 1), raw beef (2/42), ready-to-eat culinary dishes (Olivier salad, Alfredo pasta, cream puffs; 3/42), and one positive sample (1/42) of raw horse meat and one positive sample (1/42) of canned tuna. Overall, 33/42 positive samples (78.6%) originated from raw products and 9/42 (21.4%) from ready-to-eat products.

### 3.2 Phenotypic characteristics of *S. aureus* isolates

All *S. aureus* isolates obtained in this study were Gram-positive cocci and were coagulase-positive. On blood agar, the isolates demonstrated β-hemolysis, confirming their hemolytic phenotype. These phenotypic characteristics were identical for all isolates regardless of their source.

**FIGURE 2:**
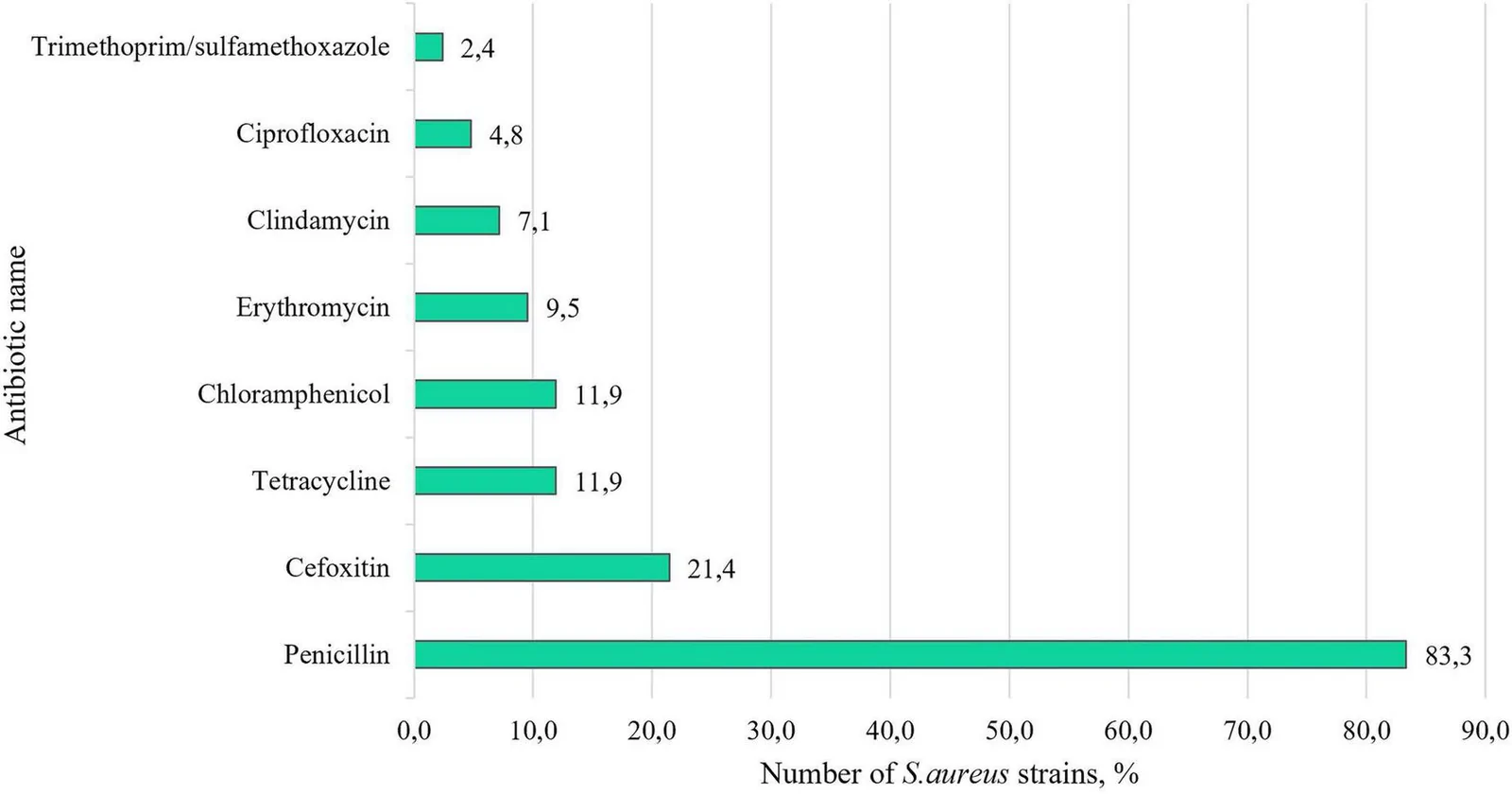
Prevalence of *S. aureus* strains resistant to different classes of antibiotics isolated from different sources (food, *n* = 42).

Antimicrobial susceptibility testing was performed for 12 antibacterial agents using the broth microdilution method. The susceptibility profiles obtained indicated variability in the levels of resistance of the isolates depending on the class of antimicrobial agents (Figure 2). At the same time, methicillin-resistant *S. aureus* (MRSA) strains were detected in 21.4% (9/42) food-derived isolates, demonstrating a positive reaction to cefoxitin (MIC >4) mg/L, while the remaining *S. aureus* strains were classified as methicillin-sensitive *S. aureus* (MSSA).

### 3.3 Molecular typing of *S. aureus* strains

**FIGURE 3:**
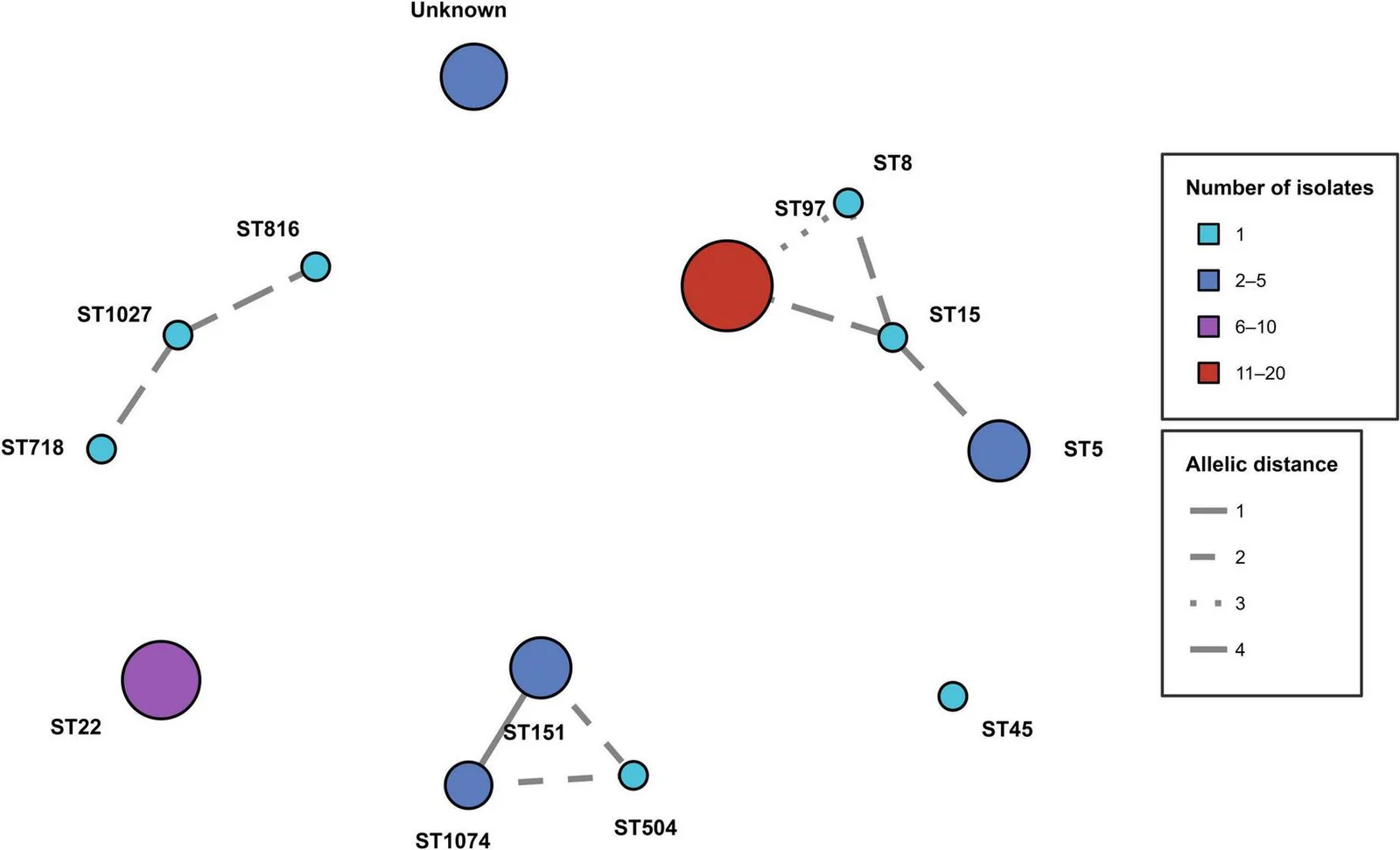
Phylogenetic tree of 42 *S. aureus* isolates based on MLST allelic profiles.

Based on the initial genome assembly-driven typing, a total of 12 distinct sequence types (STs) were formally identified among 36 complete strains, while 6 strains exhibited incomplete profiles with 6 out of 7 loci matched (Figure 3, Supplementary Table 1). As shown in this primary assembly analysis (Figure 3), the predominant sequence type was ST97 (28.57%, *n* = 12), followed by ST22 (16.67%, *n* = 7), ST5 (9.52%, *n* = 4), and ST151 (9.52%, *n* = 4). The remaining 8 sequence types – ST1074 (4.76%, *n* = 2) and ST8, ST15, ST45, ST718, ST504, ST816, and ST1027 (2.38% each, *n* = 1) – accounted for the rest of the formally assigned dataset.

Based on these primary assembly data, it was initially assumed that the unassigned strains (Sa845_KZ, Sa846_KZ, Sa847_KZ, Sa829_KZ, Sa142_KZ, and Sa2206_KZ) represented novel subtypes or variations of ST20, ST21, or ST183. Similarly, this initial screening suggested that while ST22 was heavily predominant among MRSA strains (7 out of 9 strains), the remaining two MRSA strains belonged to these unassigned variations near ST21 and ST183 (Figure 3).

However, our supplementary high-resolution SRST2 analysis successfully resolved these provisional assumptions by mapping raw reads directly against reference alleles, allowing unambiguous ST assignment for these six isolates (Supplementary Table 2). Among the MRSA strains, isolate Sa142_KZ was definitively confirmed as ST22 with an exact match at all seven loci and a sufficient average depth of 99.56×, thereby increasing the true prevalence of ST22 among MRSA to 8 out of 9 strains (88.89%). The second unassigned MRSA isolate, Sa2206_KZ, was unambiguously identified as ST398 (livestock-associated MRSA) with an exact seven-locus match and 83.27× average depth, confirming that its initial incompleteness reflected assembly gaps rather than genuine allelic novelty.

For the MSSA strains, the unresolved profiles were clarified as putative ST20 variants. Specifically, three isolates (Sa829_KZ, Sa845_KZ, and Sa846_KZ) showed an identical single-nucleotide mismatch pattern in *arcC* relative to allele 4 (SNP *arcC_4/1*) with average depths of 61.35–93.23×. This left only a single strain, Sa847_KZ, as a truly indeterminate/unresolved profile; it contained three mismatches in *arcC* relative to the closest reference allele 675 (SNP *arcC_675/3*) and could not be confidently assigned to an existing allele, corresponding to a potentially novel *arcC* allele (mean read depth of 90.79×). Complete allele data, mismatch counts, and mean read depths for all six isolates are provided in Supplementary Table 2.

### 3.4 Antimicrobial resistance determinants and regulatory genes in *S. aureus* strains

**FIGURE 4:**
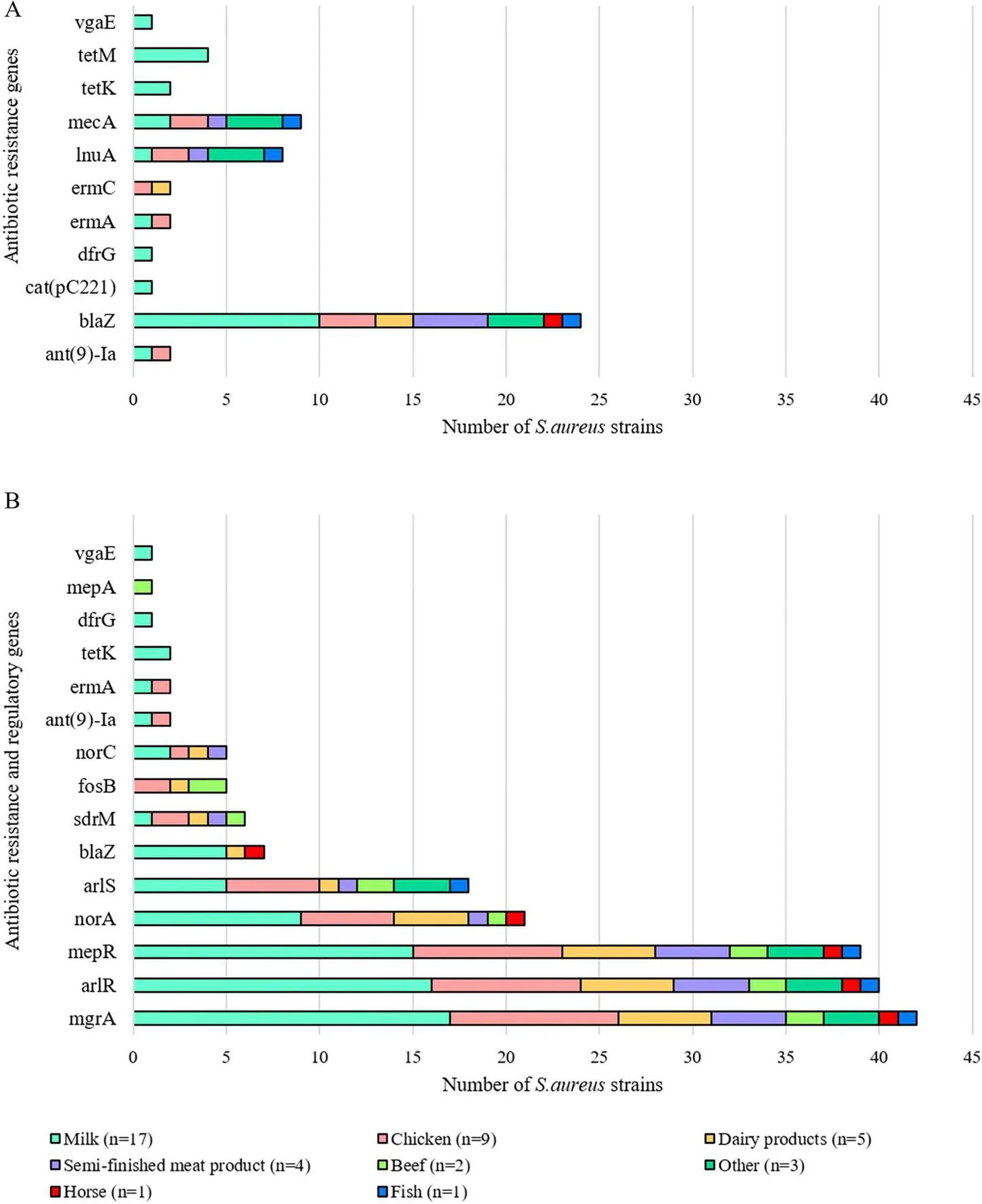
Distribution of antimicrobial resistance genes and regulatory genes among *S. aureus* strains isolated from different sources. **(A)** Genes identified using the ResFinder database; **(B)** genes identified using the CARD database. The *X*-axis shows the number of *S. aureus* strains; the color code corresponds to the source category: milk (raw cow’s and mare’s milk); dairy products (sour cream, butter); chicken meat (raw chicken fillet); beef (raw beef); horse meat (raw horse meat); fish (canned tuna); semi-finished meat products (minced beef and beef dumplings); “Other” (*n* = 3) – ready-to-eat composite culinary dishes (Olivier salad, Alfredo pasta, cream puffs). Full category definitions are given in Section “2.1 Research objects”.

*S. aureus* strains isolated from food samples showed antimicrobial resistance gene profiles (Figure 4). According to the ResFinder database, most *S. aureus* strains from food products contained genes for resistance to β-lactams (penicillin, cefoxitin) and lincosamides (clindamycin). The *blaZ* gene, encoding β-lactamase, was detected in the vast majority of strains, with the highest values in milk (10/42; 23.8%) and semi-finished meat products (4/42; 9.5%). The overall frequency of the *blaZ* gene in food-derived strains was 57.1% (24/42). Eleven isolates were phenotypically resistant to penicillin based on broth microdilution testing, whereas the *blaZ* gene was not detected by either ResFinder or CARD.

The *mecA* gene, a marker of methicillin-resistant *S. aureus* (MRSA), was detected with moderate frequency in all food categories. In milk and chicken, 4.8% (2/42) of strains were recorded. The overall frequency of *mecA* in food-derived strains was 21.4% (9/42), indicating a significant proportion of MRSA among strains isolated from food products.

The *lnuA* gene, encoding lincosamide nucleotidyltransferase, was characterized by moderate prevalence. The overall frequency of the *lnuA* gene in food-derived strains was 19% (8/42).

The *tetK* and *tetM* genes, which confer resistance to tetracyclines, showed moderate prevalence. The *tetK* and *tetM* genes were found mainly in milk, 4.8% (2/42) and 9.5% (4/42), respectively.

The *cat*(*pC221*) gene, responsible for resistance to chloramphenicol, was characterized by a low frequency in food-derived strains (1/42; 2.4%), being detected sporadically in milk (Figure 4).

Thus, milk (*n* = 17) was the main reservoir of multidrug-resistant strains, demonstrating a high frequency of *blaZ* (10 strains), *tetM* (4 strains), *tetK*, and *mecA* (2 strains each).

Two publicly available databases listed in the materials and methods section were used to analyze antibiotic resistance genes.

According to the CARD database (Figure 4), the *blaZ* gene encoding staphylococcal β-lactamase PC1 showed unexpectedly low prevalence in the study population. In strains isolated from food products, *blaZ* was detected in only 5/42 strains (11.9%), which is significantly lower than the typical prevalence of this gene among *S. aureus* (Alkuraythi et al., 2024).

In addition, Figure 4 shows the distribution of the chromosomally encoded regulatory genes *mgrA*, *arlR*, and *mepR*, which are associated with the regulation of antimicrobial resistance mechanisms and virulence.

The *mgrA* (multiple gene regulator A) gene, which encodes a global transcription regulator of the MarR family, showed widespread distribution. In food isolates, *mgrA* was detected at a frequency of 100% (42/42), indicating that the *mgrA* gene is a conserved chromosomal gene.

The *arlR* (autolysis-related locus regulator) gene, which encodes the regulatory component of the two-component ArlRS system, showed a high prevalence. In strains isolated from food products, *arlR* was detected at a frequency of 95.2% (40/42), being present in most samples. The high conservatism of *arlR* (95.7% in the entire population) emphasizes its importance for the basic physiological processes of *S. aureus.*

The *mepR* gene (multidrug efflux pump regulator), encoding the repressor of the MepA efflux pump of the MATE (multidrug and toxic compound extrusion) family, was detected with a frequency of 92.9% (39/42) of food-derived strains (Figure 4).

Regulatory genes (*mgrA*, *arlR*, *mepR*) in food-derived strains showed a high prevalence (92.9–100%) compared to the classic *blaZ* resistance gene.

**FIGURE 5:**
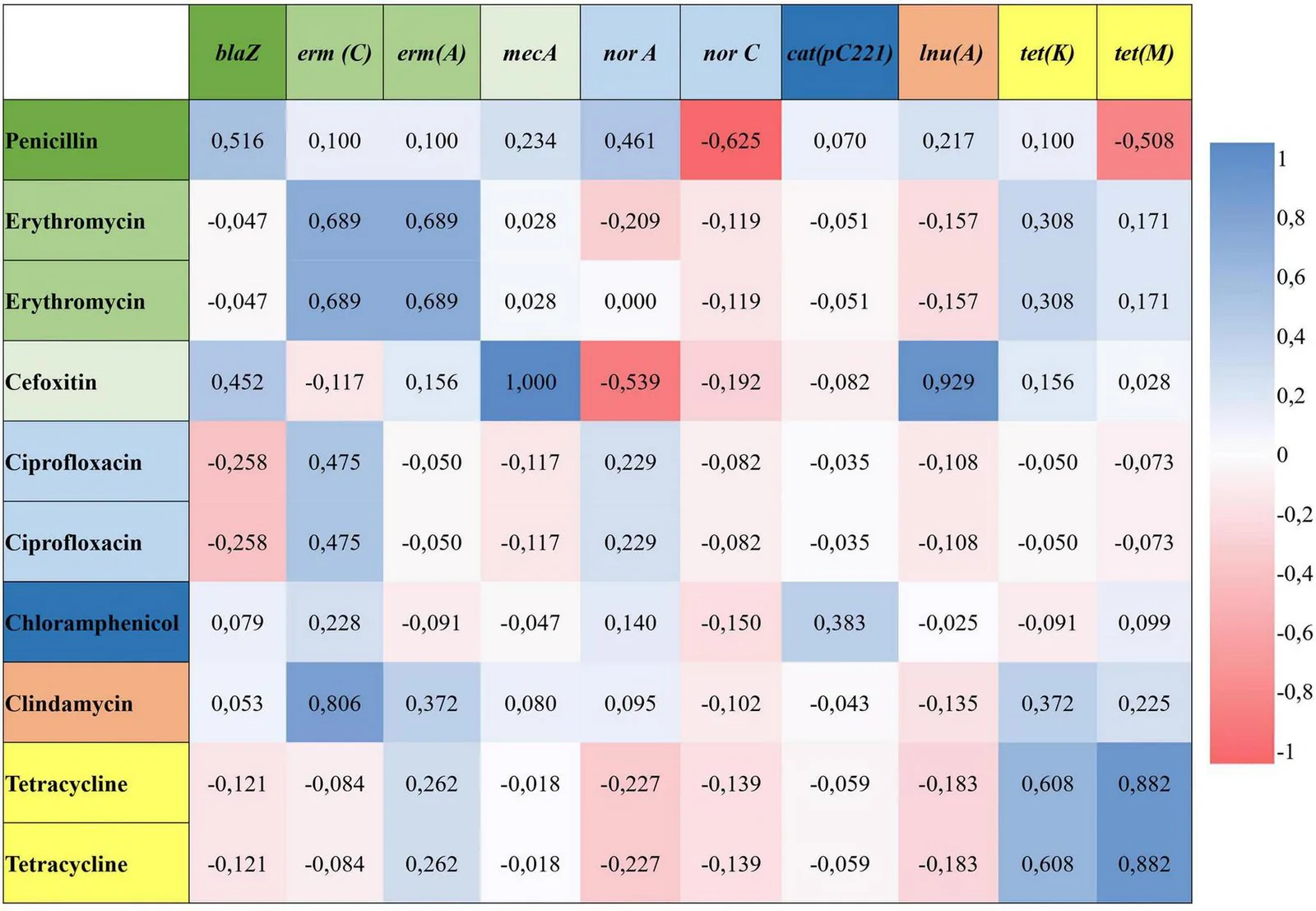
The correlation coefficient (*r*) between the demonstrated antimicrobial resistance genes in the *S. aureus* strains and different antimicrobial agents. *r* = 1 Complete (functional) positive linear correlation; 0 < *r* < 1: Positive correlation (closer to 1 – stronger, closer to 0 – weaker); *r* < 0 negative/inverse correlation.

Figure 5 shows the correlation coefficient (r) between the identified antimicrobial resistance genes in *S. aureus* strains and various antimicrobial agents.

As illustrated in Figure 5, there is a complete positive correlation between the *mecA* gene and the antibiotic Cefoxitin (*r* = 1), and a strong positive correlation is also observed between the *lnuA* gene and Cefoxitin (*r* = 0.929) and the *ermÑ* gene and the antibiotic Clindamycin (*r* = 0.806); between the *ermC* and *ermA* genes and the antibiotic Erythromycin (*r* = 0.689); a positive correlation with *r* > 0.5 is observed for Penicillin and the *blaZ* gene (*r* = 0.516), Tetracycline and the *tetK* gene (*r* = 0.608).

**FIGURE 6:**
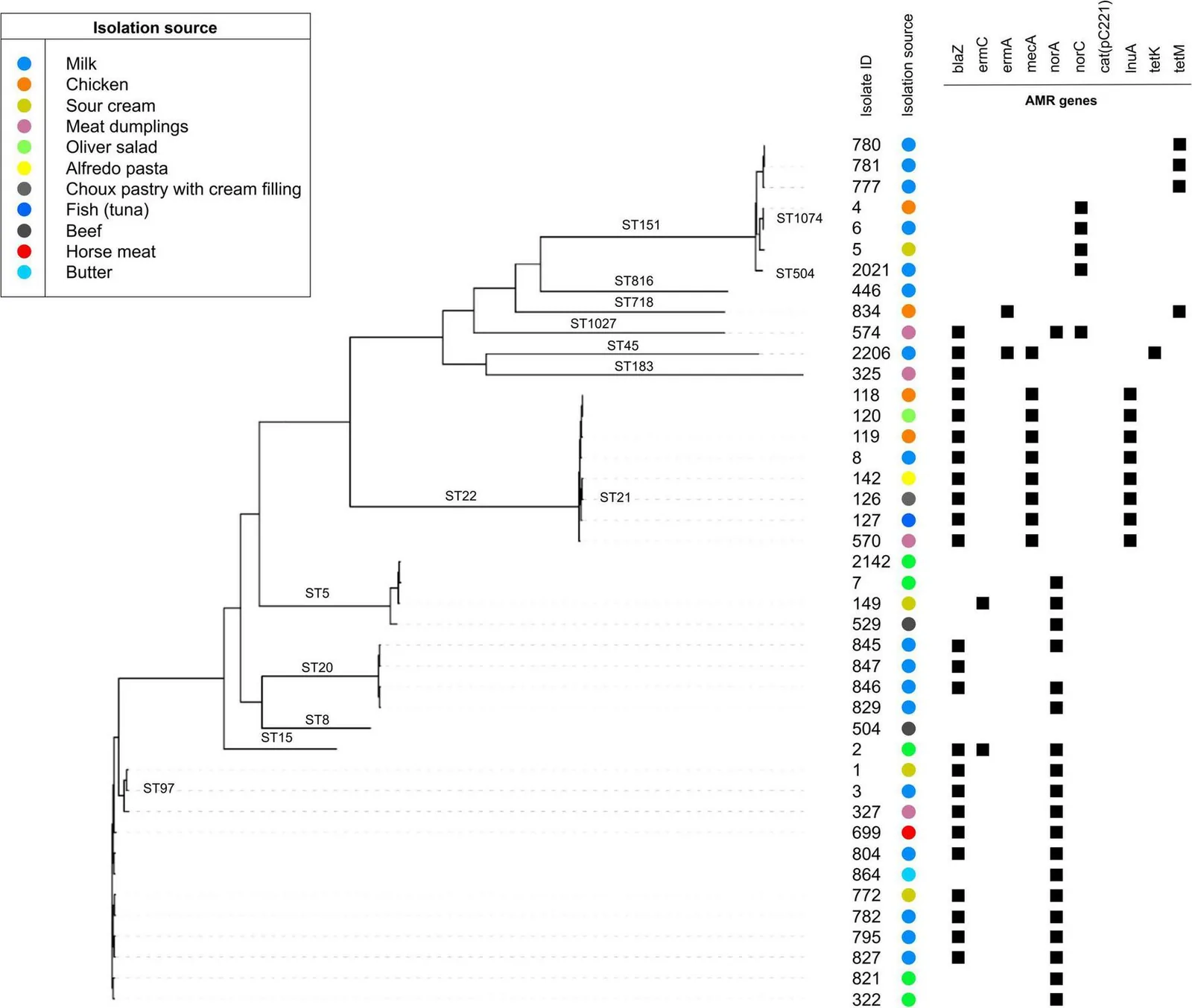
Phylogenetic tree based on core genome SNP analysis of 42 *S. aureus* strains to demonstrate the influence of antibiotic pressure based on the host.

Figure 6 demonstrates a general visualization of the resistance gene profile, the phylogenetic tree of the main genome based on SNPs, and the sources of *S. aureus* isolation.

As shown in Figure 6, subclade ST22 represents a distinct cluster of staphylococcal strains carrying a triple resistance gene profile (*blaZ*, *mecA*, and *lnuA*). All strains within this subclade were identified as MRSA. Despite belonging to the same clade, these strains were isolated from a wide variety of food samples, including both raw and ready-to-eat products, indicating a potential common source or widespread dissemination.

Prevalence of enterotoxin genes in *S. aureus* strains

**FIGURE 7:**
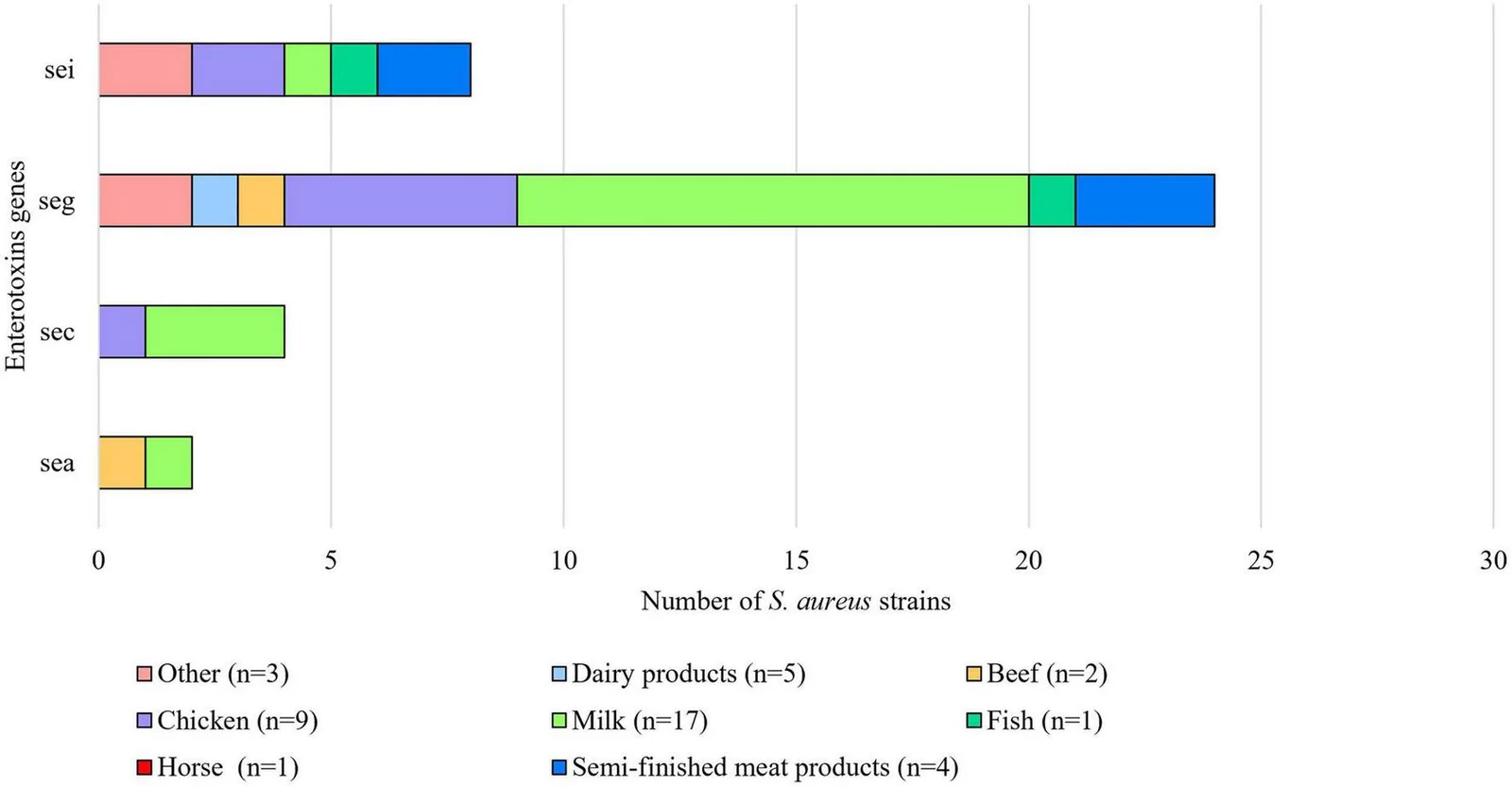
Distribution of enterotoxin genes among *S. aureus* strains isolated from different sources. The *X*-axis shows the number of *S. aureus* strains; the color code corresponds to the source category as defined in Section “2.1 Research objects” “Other” (*n* = 3) denotes ready-to-eat composite culinary dishes – Olivier salad, Alfredo pasta, and cream puffs.

**FIGURE 8:**
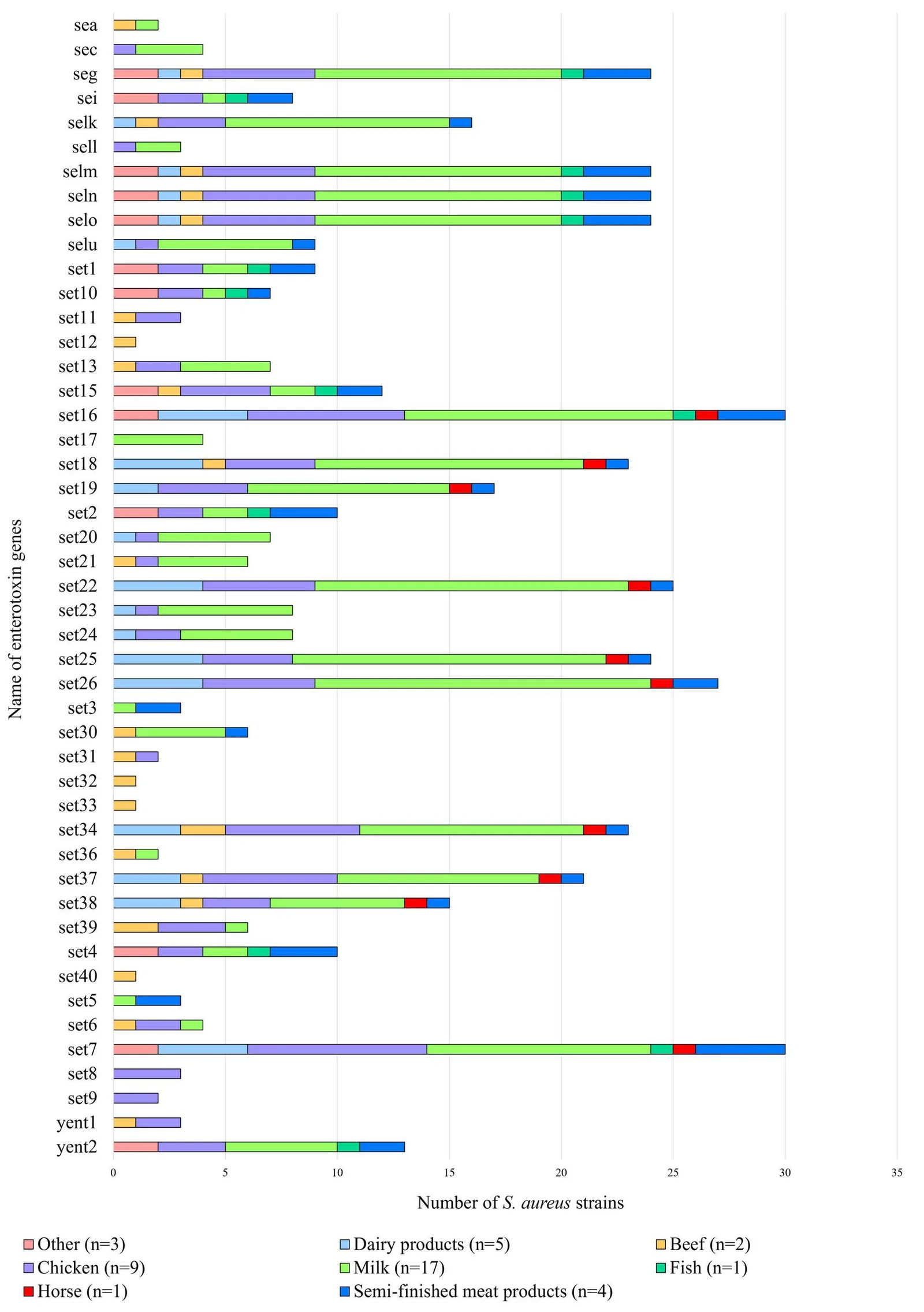
Distribution of enterotoxin – like genes among *S. aureus* strains isolated from different sources. The *X*-axis shows the number of *S. aureus* strains; the color code corresponds to the source category as defined in Section “2.1 Research objects” “Other” (*n* = 3) denotes ready-to-eat composite culinary dishes – Olivier salad, Alfredo pasta, and cream puffs.

Whole-genome sequencing revealed enterotoxin gene profiles in 42 food-derived samples (Figures 7, 8). The detected enterotoxin genes included the classical enterotoxin genes (sea and sec), the non-classical enterotoxin genes (seg and sei), and enterotoxin-like genes comprising members of the set family (set1-set13, set15-set26, set30-set34, and set36-set40), the sel genes (selk, sell, selm, seln, selo, and selu), as well as yent1 and yent2. Food-derived strains (*n* = 42) showed high variability in the number of enterotoxin genes.

Analysis of classical enterotoxin genes (Figure 7) showed that the *sea* gene was detected in 4.8% of food-derived strains (2/42). The *sec* gene was characterized by limited representation, being detected in milk in 17.6% (3/17) and in chicken in 11.1% (1/9).

Figure 7 shows that the most common among non-classical enterotoxins was the *seg* gene, showing maximum prevalence in food-derived strains at 57.1% (24/42), while the *sei* gene was detected in strains 19% (8/42) of strains.

The distribution of enterotoxin-like genes (Figure 8) was characterized by high heterogeneity and significant prevalence in food-derived strains. The most frequently occurring genes were *set7* 71.4% (30/42) and *set16* 71.4% (30/42).

Several enterotoxin-like genes showed the highest prevalence among milk-derived *S. aureus* isolates. Specifically, *set16* (12/17; 70.6%), *set18* (12/17; 70.6%), *set22* (14/17; 82.4%), *set25* (14/17; 82.4%), *set26* (15/17; 88.2%), and *seln* (11/17; 64.7%) were the most frequently detected genes in isolates recovered from milk.

Analysis of the distribution of the *yent1* and *yent2* genes (Figure 8) revealed their pronounced presence in food-derived strains. The *yent2* gene was detected in 31% (13/42) food-derived strains significantly more often than *yent1* (3/42; 7.1%). The maximum values for *yent2* were recorded in milk [76.5% (13/17)].

The yent1 gene showed a lower prevalence of 7.1% (3/42), being detected in chicken (2/9), beef (1/2). In the remaining food-derived strains, both genes were virtually absent.

A substantial proportion of *S. aureus* isolates carried multiple enterotoxin genes. The highest diversity of enterotoxin gene profiles was observed among milk-derived isolates, in which many strains harbored multiple enterotoxin-associated genes. Although the presence of these genes does not necessarily indicate toxin expression or production, it suggests the genetic potential of these isolates to produce staphylococcal enterotoxins under appropriate conditions. These findings support the need for continued microbiological surveillance of milk and dairy products to monitor the distribution of enterotoxigenic *S. aureus* strains.

### 3.5 Phenotypic characteristics of *S. aureus* strain resistance

Analysis of the antimicrobial susceptibility of *S. aureus* strains revealed a high level of resistance to penicillin: 83.3% (35/42) food-derived isolates (Supplementary Table 1).

The presence of methicillin-resistant strains in food-derived strains is particularly alarming. In this study, cefoxitin resistance was phenotypically identified in 21.4% (9/42) food-derived isolates, all of which were confirmed to harbor the *mecA* gene associated with methicillin resistance.

Resistance to other antimicrobial drugs was detected less frequently among food-derived strains and included resistance to tetracycline in 5/42 (11.9%), chloramphenicol in 5/42 (11.9%), erythromycin in 4/42 (9.5%), clindamycin in 3/42 (7.1%), ciprofloxacin in 2/42 (4.8%), trimethoprim/sulfamethoxazole in 1/42 (2.4%) (Figure 2). No resistance to gentamicin, vancomycin, linezolid, or nitrofurantoin was detected; all 42 *S. aureus* strains were susceptible to these drugs.

Among all isolated strains, 83.3% (35/42) food-derived isolates showed resistance to penicillin, with the *blaZ* gene identified in 68.6% (24/35). For these strains, the multiple antibiotic resistance (MAR) index ranged from 0.08 to 0.5 (Supplementary Table 1).

In addition, 9.5% (4/42) of food-derived strains were classified as MDR (multidrug-resistant), demonstrating resistance to at least one antimicrobial agent in three or more categories, in accordance with the criteria proposed (Magiorakos et al., 2012). Most of the MDR strains contained genes associated with antibiotic resistance and efflux systems, including *mepR*, *arlR*, *arlS*, and *mgrA*, while half of the strains contained *blaZ*, *sdrM*, *norA*, and individual strains contained *vgaE*, *ermA*, *ermC*, *ant(9)-Ia*, *dfrG*, *fosB*, *tetK*, *tetM*, and *lnuA* genes and were characterized by MAR index values ranging from 0.25 to 0.5 (Supplementary Table 1).

### 3.6 Prevalence of virulence genes, plasmid, prophages, and caspase-like systems in *S. aureus* strains

**FIGURE 9:**
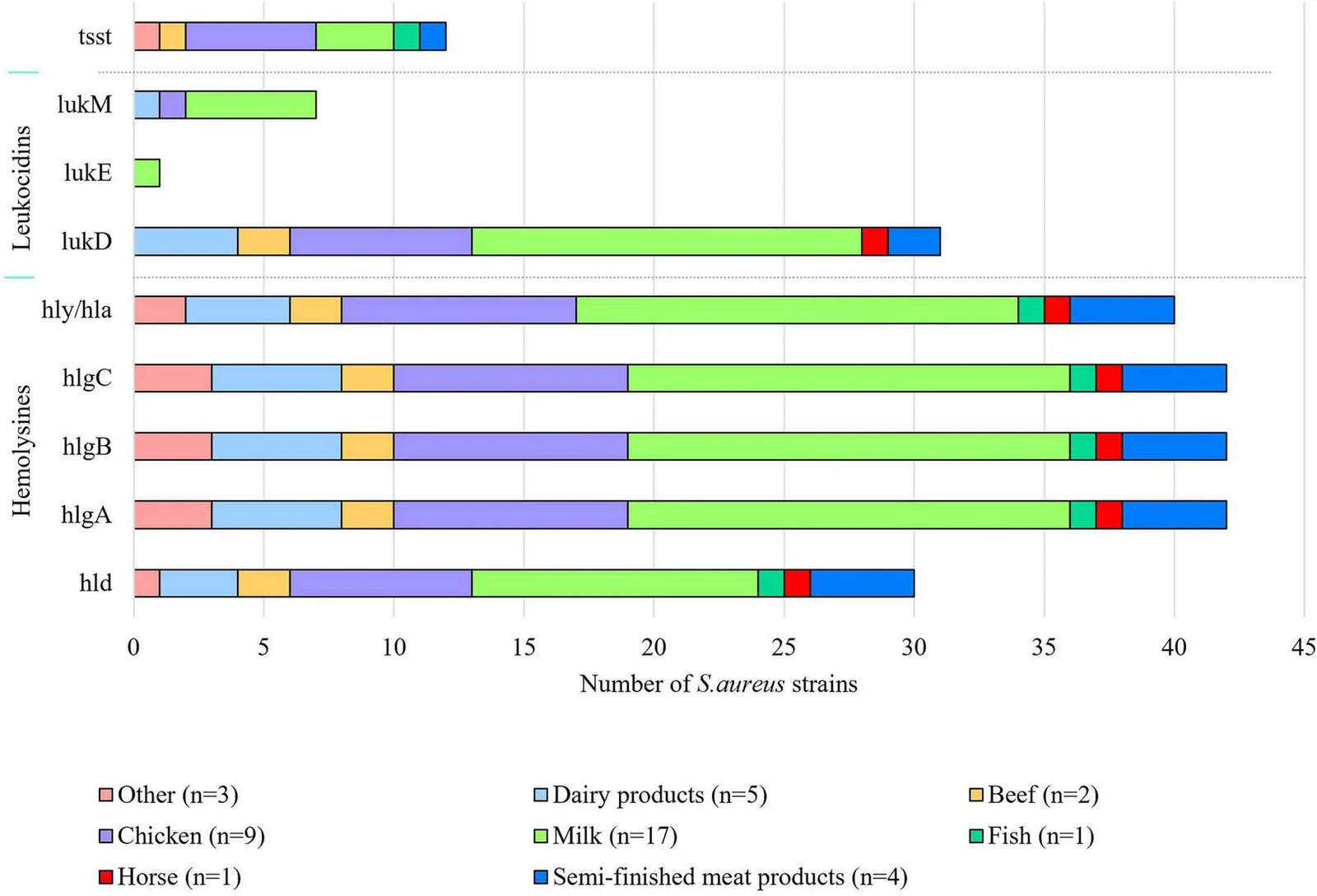
Distribution of hemolysin, leukocidin, and toxic shock syndrome toxin genes among *S. aureus* strains isolated from different sources. Toxic shock syndrome toxin gene: *tsst*; leukocidin genes: *lukD*, *lukE*, *lukM*; hemolysin genes: *hld*, *hlgA*, *hlgB*, *hlgC*, *hly/hla*. The *X*-axis shows the number of *S. aureus* strains; the color code corresponds to the source category as defined in Section “2.1 Research objects” “Other” (*n* = 3) – ready-to-eat composite culinary dishes.

The overall frequency of *tsst* gene detection (Figure 9) in food-derived strains was 28.6% (12/42), indicating the presence of *S. aureus* strains carrying the *tsst* gene in food products. The distribution of *tsst*-positive strains by food category was characterized by marked heterogeneity. The highest frequency of occurrence was recorded in chicken, 41.7% (5/12), and in milk, 25% (3/12).

Analysis of 42 food-derived *S. aureus* isolates revealed the presence of five genes encoding various hemolysins: *hld* (δ-hemolysin), *hlgA*, *hlgB*, *hlgC* (components of γ-hemolysin), and *hly/hla* (α-hemolysin) in various categories of food products (Figure 9).

The *hly/hla* gene, encoding α-hemolysin (α-toxin), showed the highest prevalence among all hemolysin genes studied in food-derived strains and was characterized by maximum variability across product categories.

Analysis of food-derived strains presented 100% occurrence of *hlgA, hlgB, and hlgC* genes. Milk (*n* = 17) showed an absolute maximum with detection of *hlgA, hlgB, hlgC*, and *hly/hla* in 100% of strains (17/17), indicating the universal presence of this key virulence factor in all strains isolated from milk.

Milk-derived isolates most frequently carried a complete repertoire of hemolysin genes. Although the presence of these genes does not necessarily indicate toxin expression or pathogenicity, it suggests that these isolates possess the genetic potential to produce multiple hemolysins under appropriate conditions.

The leukocidin-encoding genes *lukD*, *lukE*, and *lukM* are important virulence factors of *S. aureus*, determining its ability to damage leukocytes and evade the host’s immune response.

Analysis of the distribution of leukocidin-encoding genes among food-derived strains revealed marked differences in their frequency of isolation. In food-derived strains, the *lukD* gene was detected in 73.8% (31/42). The highest number of positive strains was found in milk [35.7% (15/42)] and chicken meat [16.7% (7/42)], indicating significant circulation of this virulence factor in these food niches (Figure 9).

The *lukE* gene was found sporadically and was detected in only one strain isolated from milk, reflecting its extremely low prevalence in the study strains.

Among the food-derived isolates, only 2.4% (1/42) simultaneously harbored both *lukD* and *lukE* genes. These genes encode the two components of the bicomponent LukDE leukocidin, a virulence factor involved in the lysis of host leukocytes and immune evasion. Although the presence of *lukD* and *lukE* indicates the genetic potential to produce LukDE, the expression and biological activity of this leukocidin were not evaluated in the present study.

At the same time, the *lukM* gene was detected in 11.9% (5/42) of strains isolated from milk, as well as in 2.4% (1/42) of strains isolated from dairy products and chicken meat, indicating its moderate but stable circulation in various types of food raw materials and products.

The data obtained indicate the dominance of the *lukD* gene in strains isolated from products, as well as a significantly lower prevalence of *lukE* and *lukM*.

The results obtained indicate marked heterogeneity in the distribution of leukocidin-encoding genes among food strains of *S. aureus.*

We note that while short-read Illumina sequencing and reliance on database-defined replicons pose inherent limitations for plasmid identification, the consistent lack of plasmid markers across all 42 genomes during the annotation pipeline analysis indicates a likely chromosomal configuration for *blaZ*, *mecA*, and *lnuA*. Therefore, horizontal dissemination via classical plasmid transfer appears less probable in this collection.

In this study, 42 *S. aureus* strains were analyzed using the CRISPRCasFinder database. No confirmed CRISPR loci were identified in any of the investigated isolates. However, Cas3 Type I CRISPR-associated proteins were detected in 28.6% (12/42), and their distribution among the different food categories was not statistically significant (*p* > 0.05).

The prophage content of 42 *S. aureus* strains was systematically assessed using the PHASTEST web server. Analysis revealed that 37 of the 42 strains examined harbored at least one integrated prophage element, while the remaining five strains were devoid of detectable prophage sequences. Among the prophage types identified, PHAGE_Staphy_DW2 (NC_024391), PHAGE_Staphy_vB_SauS_phi2 (NC_028862), and PHAGE_Staphy_JS01 (NC_021773) exhibited the highest prevalence (Supplementary Table 1). The widespread distribution of prophage elements across the analyzed strain collection is indicative of a substantial contribution of horizontal gene transfer to the genomic plasticity and diversification of food-associated *S. aureus* populations; however, an in-depth characterization of prophage genomic organization and its associated functional implications fell outside the primary objectives of the present study.

## 4 Discussion

To our knowledge, this is the first whole-genome-based characterization of *S. aureus* from the food chain of Northern Kazakhstan - a region largely absent from global genomic surveillance despite lying at the crossroads of European, Russian, Chinese and South Asian livestock and trade corridors. The recovery of 42 isolates from 181 food samples establishes *S. aureus* as a persistent rather than incidental occupant of the local food supply.

Among the 42 food-derived *S. aureus* isolates, the largest number originated from raw milk (17/42; 40.5%). Considering the total number of samples analyzed, *S. aureus* was detected in 17 of 38 raw milk samples (44.7%), a finding congruent with pooled international estimates of 33.36% prevalence in raw milk (Zhang et al., 2022), 28.4% across dairy products (Liang et al., 2023), and 11.6% for enterotoxigenic strains in raw milk (Shalaby et al., 2024). Raw milk, therefore, remains the single most consequential entry point for this pathogen into the human diet. Contamination of chicken meat was likewise consistent with the 29.2% pooled prevalence reported for raw meat by Ou et al. (2017), whose subgroup analysis showed the highest chicken contamination in Asian studies, a significant decline over time, and lower rates in European products. Beef and horse meat, by contrast, were only sporadically contaminated, against a beef prevalence of 23.8% in Iran, 27.8% in the United States, and 24.5% across African countries (Alkuraythi et al., 2024), and 19.7% among retail meat in Shandong, China (Zhao et al., 2025). Literature on horse meat is essentially absent given its culturally restricted consumption; we have previously characterized a single horse-meat isolate in detail (Kozhakhmetova et al., 2025). A single tuna isolate, set against 15% prevalence in Indian seafood (Sivaraman et al., 2022), is insufficient for inference but establishes that retail fish is not exempt. Contamination of semi-finished meat products fell well below the 35.7% pooled estimate for processed meat (Liang et al., 2023) and is most parsimoniously explained by carry-over from raw material. Collectively, these matrix-specific differences reflect hygiene practice, storage, transport conditions, and temperature conditions.

The resistome was dominated by *blaZ*, the expected signature of sustained penicillin use in veterinary practice; comparable frequencies are 70% in meat in Saudi Arabia (Alkuraythi et al., 2024), while Pennone et al. (2022), interrogating 29,679 genomes, located *blaZ* on plasmid contigs in 30% of cases. Alkuraythi et al. (2024) have emphasized that determinants shared between food and clinical isolates raise the possibility of transfer from the food chain into clinical settings. The marked discrepancy in *blaZ* detection between CARD and ResFinder is methodological rather than biological: CARD applies stringent identity thresholds suited to mechanistic classification, allowing divergent alleles to escape detection, whereas ResFinder targets acquired determinants with more permissive cut-offs and higher sensitivity – a behavior well recognized in the literature (Bortolaia et al., 2020) – and ResFinder was therefore adopted as primary.

Consistent with this observation, phenotypic resistance to penicillin was detected in 11 isolates in which the *blaZ* gene was not identified by either CARD or ResFinder. Such phenotypic–genotypic discordance has been reported previously and may be explained by sequence variation within *blaZ*, alternative β-lactam resistance mechanisms, differences in gene expression, or limitations of current resistance gene databases. Because additional confirmatory testing, such as PCR targeting *blaZ* or β-lactamase activity assays, was not performed, the molecular basis of penicillin resistance in these isolates could not be elucidated and warrants further investigation.

No plasmid replicons were detected, indicating that *blaZ*, *mecA*, and *lnuA* are predominantly chromosomally encoded in this collection. This attenuates but does not eliminate concern over horizontal transfer, since genomic islands, bacteriophages, insertion sequences, and transposons cannot be excluded from short-read data; what can be inferred is that the rapid dissemination characteristic of broad-host-range plasmids is unlikely here. The *mecA* frequency indicates a substantial MRSA fraction, sitting mid-range within a highly heterogeneous global distribution: MRSA was reported in 68.8% of 186 publications on milk and dairy products (González-Machado et al., 2024), with national estimates from 0.29% (United Kingdom) and 8.1% (Portugal) to 14% (Mexico) and 75.4% (Turkey), alongside a substantial African literature (Titouche et al., 2022). Among minor determinants, *ant(9)-Ia* is rarely reported in foodborne *S. aureus*, and its presence in several MDR isolates may signal exchange between strains of different ecological origin; *tetK* was recovered at a frequency close to the 4% reported in retail meat (Alkuraythi et al., 2024); and *lnuA* was more prevalent than in comparable collections, where it has been rare (Szczuka et al., 2022).

The regulatory genes *mgrA*, *arlR* and *mepR* were universal, consistent with their conserved chromosomal nature and with comparative genomic data (Guo et al., 2024) and the ranking of *arlR* second and *mepR* fifth among the most frequent genes across 95 genomes (Edet et al., 2025).

MLST revealed a heterogeneous population whose composition differs appreciably from that of neighbouring regions – an argument in itself for national-level surveillance. Yang et al. (2018) found ST6 dominant among ready-to-eat foods across 24 Chinese cities, with all ST6 isolates carrying *sea*; ST6 was absent here. Ning et al. (2023) resolved raw-milk isolates from Hunan into 22 sequence types, with ST7 most frequent; Mechesso et al. (2021) recovered only ST72 and ST398 from Korean goats, raising the prospect of caprine-to-human MRSA transmission; and Pasquali et al. (2022) reported ST121, ST398, ST5, ST1, ST7 and ST15 in Mediterranean artisanal animal products, singling out ST121 as virulent. Mikhaylova et al. (2022) distributed isolates from Russian ready-to-eat foods across 15 sequence types with ST22 at a frequency nearly identical to ours, while ST97 – dominant in our collection – was absent. ST97 is a bovine-adapted, mastitis-associated genotype capable of animal-to-human transmission and carrying enterotoxin and cytotoxin genes, circulating in China, Spain, Japan, the Netherlands, and Brazil (Jolley et al., 2018; Fursova et al., 2020). ST151 is likewise a leading cause of bovine mastitis with substantial economic consequences (Sung and Lindsay, 2007). ST22 is widely recovered from food, often as ST22-MRSA, and implicated in multidrug-resistant foodborne infection (Zhao et al., 2023), while ST5 shows high adaptability, frequent methicillin resistance and enterotoxin production (Jiang et al., 2025).

Staphylococcal enterotoxins resist heat and low pH (Necidová et al., 2019) and gastrointestinal proteolysis (Pinchuk et al., 2010), remaining active after the producing cells are killed – the property that makes them central to food safety. Reported profiles vary widely: *sep*, *seg*, *sei*, *sem*, *sen*, *seo* and *selu* each occurred in 33.73% of Chinese food isolates (Chen et al., 2023); *sei*, *seg*, *seln* and *selm* were found in US chicken isolates (Faraj et al., 2025); *sea* predominated in Iran (45.5%), followed by *sed* (36.4%) and *seg* (18.2%) (Forouzani-Moghaddam et al., 2024); and *selX* reached 94.8% in Saudi Arabia (Alkuraythi et al., 2024). Our profile was characterized by the predominance of non-classical *seg* and *sei* over classical types, particularly among milk isolates. Structurally, SEC and SEG possess a single low-affinity MHC II α-chain binding site, whereas SEA and SEI carry an additional high-affinity β-chain site correlating with stronger superantigenic activity; SEA, SEC, and SEG bear a disulfide-stabilized cystine loop (Fisher et al., 2018) that SEI lacks, an element considered critical for emetic activity (Cieza et al., 2024). Both *seg* and *sei* belong to the *egc* operon, increasingly implicated in staphylococcal food poisoning even without classical enterotoxins: Omoe et al. (2005) showed that *egc* genes are actively transcribed and may be the sole enterotoxin genes present, and Zhao et al. (2023) identified strains carrying the complete operon without classical SEs. Because commercial diagnostic kits target classical toxins almost exclusively, genetic screening remains indispensable for exposing hidden toxigenic risk in the dairy chain. The high frequency of the enterotoxin-like genes *set7* and *set16* broadens this superantigenic repertoire further, although expression and thermostability were not validated phenotypically.

*Staphylococcus aureus* deploys a redundant virulence arsenal in which functionally overlapping factors preserve fitness when any single one is neutralized (Tam and Torres, 2019). The near-universal distribution of haemolysin genes in our collection parallels that in raw Chinese shrimp (Dai et al., 2023) and Russian ready-to-eat products (Mikhaylova et al., 2022); Alkuraythi et al. (2024) recovered *hly/hla*, *hlgA/hlgB* and *hld* from all isolates, designating them key virulence genes, and their lytic action on erythrocytes, platelets and lipid-rich membranes is well characterized (Sivaraman et al., 2022). Of greater epidemiological consequence is *tsst*, encoding a protease- and heat-stable superantigen that promotes persistence across ecological niches (Touaitia et al., 2025) and precipitates toxic shock syndrome, whose severity is compounded by accessory virulence factors (Govari and Pexara, 2025); TSST-1-producing strains cluster within CC5 and ST22 (Touaitia et al., 2025). Our prevalence exceeds that reported in raw Chinese shrimp (3.2%; Dai et al., 2023) and Mongolian raw beef (17.1%; Dorjgochoo et al., 2023), while coinciding with the 28.6% in Russian ready-to-eat foods, where *tsst* was chiefly linked to ST22-MRSA (Mikhaylova et al., 2022). The *lukD* frequency is consistent with the estimate that *lukED* occurs in roughly 70% of strains and is maintained lineage-specifically (Tam and Torres, 2019), though lower than the 94.3% reported in US bulk tank milk (Patel et al., 2021); its epidemiology is not static, as Lu et al. (2024) documented a decline from above 84% to 63.3% between 2012 and 2021 in eastern China. Finally, *lukM*, largely restricted to bovine clinical mastitis and rarely reported in food microbiology, is highly cytotoxic to ruminant neutrophils and contributes to intramammary infection (Vrieling et al., 2015; Hoekstra et al., 2018); its presence further indicates a dairy-herd reservoir feeding the food chain.

The most consequential finding is an ST22-MRSA clone carrying a combined resistance–hypervirulence profile (*blaZ*, *mecA*, *lnuA* with *tsst-1* and the *egc* cluster). ST22 (EMRSA-15) is among the most widely disseminated epidemic lineages globally, and its establishment in the food supply represents a shift in ecological niche; high colonization capacity together with *tsst-1* and *egc* co-carriage defines a superantigenic profile capable of causing severe, systemically mediated foodborne illness. The near-identity of *tsst* prevalence with Russian ready-to-eat foods (Mikhaylova et al., 2022), against lower values in China (Dai et al., 2023) and Mongolia (Dorjgochoo et al., 2023), may point to a shared reservoir across the post-Soviet food-supply chain – a hypothesis requiring multi-center verification. The clonal landscape (ST97 > ST22 > ST5) also diverges from East Asian and Western European profiles, suggesting a distinct Central Asian population structure. These inferences must be weighed against the study’s limitations: the sample size restricts precision and precludes formal comparison between matrices; exclusive reliance on short-read Illumina sequencing prevents definitive resolution of plasmid architecture and the genomic context of mobile elements, so that contributions of insertion sequences or transposons cannot be excluded; enterotoxin expression was not confirmed phenotypically; and the mechanism of penicillin resistance in *blaZ*-negative isolates remains undetermined. Long-read sequencing (Oxford Nanopore) of selected MRSA ST22 isolates carrying the triple resistance profile (*blaZ, mecA, lnuA*) is planned for future work.

## 5 Conclusion

This study provides the first genomic characterization of 42 food-borne *S. aureus* isolates from Northern Kazakhstan and, to our knowledge, from Central Asia as a whole. While the sample size is limited, the results offer preliminary insights that help address a recognized geographic gap in the global AMR and virulence surveillance landscape.

The identification of an ST22-MRSA clone harboring a combined resistance-hypervirulence profile (*blaZ-mecA-lnuA* with *tsst-1* and the *egc* enterotoxin cluster) suggests that internationally significant epidemic lineages circulate in the Northern Kazakhstani food chain. The *tsst* prevalence in food-derived isolates (28.6 %) appears notably higher than in most international food surveys and closely resembles data from Russia, which may indicate a shared post-Soviet epidemiological corridor, although this observation requires confirmation with larger cohorts. The clonal structure observed (ST97 > ST22 > ST5) differs from those reported in China, Western Europe, and Korea, highlighting the potential importance of region-specific genomic surveillance. The predominance of non-classical enterotoxin genes (*seg, sei*) over classical types points to a possible diagnostic blind spot in current food-safety screening panels that rely exclusively on *sea-see* detection.

These preliminary findings support the rationale for integration of WGS-based typing into the national food-safety surveillance system of Northern Kazakhstan, with particular attention to MRSA-ST22 lineage tracking in dairy and poultry supply chains, expansion of routine enterotoxin screening panels to include *egc*-cluster genes and *tsst*-1, and exploration of bilateral data-sharing frameworks with neighboring countries to enable monitoring of transboundary AMR clone dissemination across Central Asia.

The present study provides local genomic data that may contribute to future regional surveillance efforts to monitor the molecular epidemiology of food-borne *S. aureus* in Central.

## Data Availability

The datasets presented in this study can be found in online repositories. The names of the repository/repositories and accession number(s) can be found in the article/Supplementary material.

## Footnotes

1 https://www.eucast.org/bacteria/methodology-and-instructions/disk-diffusion-and-quality-control/.

2 https://www.bioinformatics.babraham.ac.uk/projects/fastqc/.

3 https://pubmlst.org/organisms/staphylococcus-aureus.

4 https://card.mcmaster.ca/.

5 https://genepi.food.dtu.dk/resfinder.

6 https://www.mgc.ac.cn/VFs/.
