# Supplementary material for: Whole-genome analysis and phenotypic antimicrobial resistance of Staphylococcus aureus isolated from foods in Northern Kazakhstan
Source: Front Microbiol. 2026 Aug 7;17:1910820. doi: 10.3389/fmicb.2026.1910820 (PMC13493241; doi:10.3389/fmicb.2026.1910820)
Supplement: Supplementary file 1 [file Table_2.docx]

**Supplementary Material**

**Table S2** Allele assignment by SRST2-based MLST for the six isolates with incomplete profiles obtained from genome assembly.

| **Isolate** | **ST** | ***arcC*** | ***aroE*** | ***glpF*** | ***gmk*** | ***pta*** | ***tpi*** | ***yqiL*** | **Mismatches** | **Mean depth (×)** |
| --- | --- | --- | --- | --- | --- | --- | --- | --- | --- | --- |
| Sa829 | putative 20* | 4* | 9 | 1 | 8 | 1 | 10 | 8 | *arcC*_4/1 SNP | 61.35 |
| Sa142 | 22 | 7 | 6 | 1 | 5 | 8 | 8 | 6 | 0 | 99.56 |
| Sa845 | putative 20* | 4* | 9 | 1 | 8 | 1 | 10 | 8 | *arcC*_4/1 SNP | 69.36 |
| Sa846 | putative 20* | 4* | 9 | 1 | 8 | 1 | 10 | 8 | *arcC*_4/1 SNP | 93.23 |
| Sa847 | undetermined / unresolved ST* | 675* | 9 | 1 | 8 | 1 | 10 | 8 | *arcC*_675/3 SNP | 90.79 |
| Sa2206 | 398 | 3 | 35 | 19 | 2 | 20 | 26 | 39 | 0 | 83.27 |

ST, sequence type; SNP, single-nucleotide polymorphism. The seven columns *arcC*–*yqiL* are the housekeeping loci of the *S. aureus* MLST scheme.

* Provisional assignment. Sa829, Sa845 and Sa846 were resolved as putative ST20 variants, each carrying a single mismatch in *arcC* relative to allele 4 (*arcC*_4/1 SNP). Sa847 remained unresolved, with three mismatches in *arcC* relative to allele 675 (*arcC*_675/3 SNP), indicating a potentially novel allele.

Mismatches, locus and number of single-nucleotide differences from the closest reference allele; 0 indicates an exact seven-locus match. Mean depth, mean read depth (×) across the seven loci.
